# Unexpected Ground-State Structure and Mechanical Properties of Ir_2_Zr Intermetallic Compound

**DOI:** 10.3390/ma11010103

**Published:** 2018-01-10

**Authors:** Meiguang Zhang, Rui Cao, Meijie Zhao, Juan Du, Ke Cheng

**Affiliations:** 1 College of Physics and Optoelectronics Technology, Baoji University of Arts and Sciences, Baoji 721016, China; RuiCaobj@126.com (R.C.); MeijieZhao1@126.com (M.Z.); JuanDub@126.com (J.D.); 2 College of Optoelectronic Technology, Chengdu University of Information Technology, Chengdu 610225, China; ck@cuit.edu.cn

**Keywords:** first-principles calculations, ground-state structure, Ir_2_Zr intermetallic, mechanical properties

## Abstract

Using an unbiased structure searching method, a new orthorhombic *Cmmm* structure consisting of ZrIr_12_ polyhedron building blocks is predicted to be the thermodynamic ground-state of stoichiometric intermetallic Ir_2_Zr in Ir-Zr systems. The formation enthalpy of the *Cmmm* structure is considerably lower than that of the previously synthesized Cu_2_Mg-type phase, by ~107 meV/atom, as demonstrated by the calculation of formation enthalpy. Meanwhile, the phonon dispersion calculations further confirmed the dynamical stability of *Cmmm* phase under ambient conditions. The mechanical properties, including elastic stability, rigidity, and incompressibility, as well as the elastic anisotropy of *Cmmm*-Ir_2_Zr intermetallic, have thus been fully determined. It is found that the predicted *Cmmm* phase exhibits nearly elastic isotropic and great resistance to shear deformations within the (100) crystal plane. Evidence of atomic bonding related to the structural stability for Ir_2_Zr were manifested by calculations of the electronic structures.

## 1. Introduction

The platinum-group metals (PGMs)—osmium, iridium, platinum, ruthenium, rhodium, and palladium—are immensely important in numerous technologies, but the experimental and computational data on their binary alloys still contain many gaps. In contrast to other transition metals [1,2,3], interest in PGMs is driven by their essential role in a wide variety of industrial applications, which is at odds with their high cost. The primary application of PGMs is in catalysis, where they are core ingredients in the chemical, petroleum, and automotive industries. Recently, the Ir-Zr binary alloys have become a topic that is currently attracting considerable interest for their potential applications in aeronautics and electronics applications. Like Ni-based alloys, Ir-Zr alloys have a two-phase face-centered cubic (*fcc*)/L1_2_ structure, with the L1_2_ (Ir_3_Zr) precipitates coherently embedded in the *fcc* Ir matrix, and such an alloy with *fcc*/L1_2_ interfaces has been found to have higher strength than the single *fcc* or L1_2_ intermetallic [4,5,6,7,8]. Moreover, it has been demonstrated that, for a given alloy, some unexpected stoichiometric intermetallics would appear in its microstructure during different heating and cooling processes, which would have a great effect on the properties of the alloys [9,10,11]. For instance, it has been reported that the phase transformation of IrZr significantly contributes to the great high-temperature shape memory effect for Ir-Zr alloys [10,11]. Hence, an insight into the intrinsic properties of the intermetallics, such as their mechanical properties, could be of great practical significance for the design of composite materials.

The Ir-Zr binary system has been reviewed by Okamoto [12], where the phase diagram was based primarily on the high-temperature X-ray and thermal analyses conducted by Eremenko et al. [13]. Six known stoichiometric intermetallics—Ir_3_Zr, Ir_2_Zr, IrZr, Ir_3_Zr_5_, IrZr_2_, and IrZr_3_—have been determined, in which Ir_3_Zr and IrZr melted congruently, and the others were formed by peritectic or peritectoid reactions. The follow-up studies on their structures, mechanical, thermodynamic, and dislocation properties have evoked significant interest in their potential applications. It is known that crystal structures are the key for understanding the mechanical properties of materials [14,15,16,17,18]. Thus, the crystal structures of these six intermetallic compounds have been extensively studied. Except for IrZr and Ir_2_Zr, consensus has been reached that the intermetallic phases Ir_3_Zr, Ir_3_Zr_5_, IrZr_2_, and IrZr_3_ adopt cubic Cu_3_Au-type ($Pm\bar{3}m$, Z = 1), hexagonal Mn_5_Si_3_-type (*P*6_3_*/mcm*, Z = 2), tetragonal Al_2_Cu-type (*I*4/*mcm*, Z = 4), and tetragonal V_3_S-type ($I\bar{4}m2$, Z = 8) structures, respectively. For IrZr, different room-temperature structures, including TiNi-type, FeB-type, and CrB-type, have been proposed, while a new orthorhombic *Cmcm* structure was experimentally characterized to be the most stable phase for IrZr [15], and was confirmed by the theoretical work in [10,16,18]. However, the crystal structure of Ir_2_Zr is the subject of continuing debate. Ir_2_Zr was proposed to have the *C*15 (Cu_2_Mg-type) structure [13], while recently, it was theoretically suggested to be unstable through formation enthalpy calculations [18]. Compared to IrZr, experimental and theoretical investigation of the crystal structures of Ir_2_Zr have rarely been undertaken, and there is a lack of confirmed reports on the existence of ground-state structure. Therefore, the peculiarity and the absence of characterized stable structures of Ir_2_Zr prompted our endeavor to investigate its structural stability under ambient conditions. Furthermore, the explorations of ground-state structures and related mechanical properties would provide more insights on other Ir-based intermetallic compounds. In order to address these points, we here performed extensive structure searches to explore the potential energetically stable Ir_2_Zr phase at ambient pressure using the newly developed Crystal structure AnaLYsis by Particle Swarm Optimization package (CALYPSO) [19,20], unbiased by any known information. This method has been successfully applied to extensive structures that have been confirmed by independent experiments [21,22,23]. Indeed, an orthorhombic *Cmmm* structure is uncovered to be the best ground-state candidate for Ir_2_Zr, and the crystal structures, mechanical behaviors, and electronic structures of this new phase were then fully investigated in comparison with the proposed Cu_2_Mg-type phase. 

## 2. Computational Methods

The crystal structure searches for Ir_2_Zr were performed based on a global minimization of energy surfaces merging first-principle total-energy calculations as implemented in CALYPSO code [19,20], which was designed to predict stable or metastable crystal structures requiring only chemical compositions of a given compound at given external conditions (e.g., pressure). Here, using the CALYPSO code in combination with Vienna ab initio simulation package (VASP) [24], variable cell structure searches for Ir_2_Zr containing 1–6 formula units (f.u.) in the simulation cell were systematically performed at ambient pressure. During the structure searches, the 60% of the structures of each generation with the lowest enthalpies were selected to generate the structures for the next generation by Particle Swarm Optimization (PSO) operation, and the other structures in new generation were randomly generated to increase the structural diversity. The following local structural relaxations and electronic calculations were performed using the VASP code, in which the generalized-gradient approximation proposed by Perdew-Burke-Ernzerhof exchange-correlation functional [25,26] was used for the full optimization of all crystal structures. The electron and core interactions were included by using the frozen-core all-electron projector augmented wave potential of the metal atoms including d electrons as valence states [27]. The cutoff energy of 600 eV for the plane-wave expansions and dense *k*-point with grid density of 0.03 × 2π Å^−1^ (Monkhorst-Pack scheme) [28] were used in the Brillouin zone integration. The total energy and stress calculations were performed by using the tetrahedron method with Blöch corrections and Gaussian smearing method, respectively. The structural relaxation was performed using the conjugate gradient method until total energy is converged to within 10^−5^ eV and the force on each atom is less than 0.01 eV∙Å^−1^. The phonon spectra of the *Cmmm* structure was calculated by the finite displacement method, which is based on first-principles calculations of total energy, Hellman-Feynman forces, and the dynamical matrix as implemented in the PHONOPY package [29]. The independent single crystal elastic constants were determined from evaluation of stress tensor generated small strain (stress-strain approach) [30], and the polycrystalline elastic moduli including bulk modulus, shear modulus and Young’s modulus, as well as Poisson’s ratio, were thus estimated by the Voigt-Reuss-Hill approximation [31].

## 3. Results and Discussion

**Figure 1:**
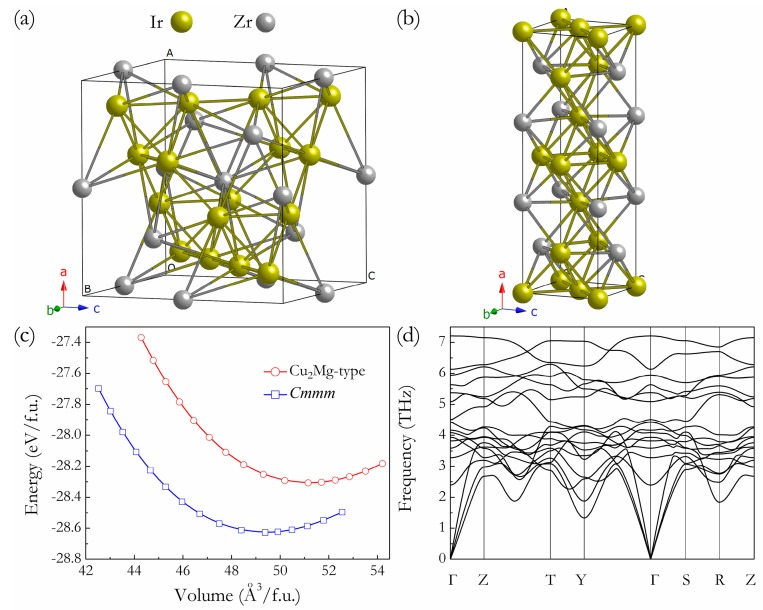
Crystal structures of Cu_2_Mg-Ir_2_Zr (**a**) and *Cmmm*-Ir_2_Zr (**b**), the large and small spheres represent Ir and Zr atoms, respectively. The total energy vs. f.u. volume for Cu_2_Mg-type and *Cmmm* structures (**c**) and phonon dispersion curves of *Cmmm*-Ir_2_Zr at 0 GPa (**d**).

Under ambient conditions, the structure searches with the only input being the chemical composition of Ir:Zr = 2:1 predicted the most stable structure to be the orthorhombic *Cmmm* structure containing four f.u. per unit cell, as presented in Figure 1b, together with the previous experimental Cu_2_Mg-type phase Figure 1a. Compared to the building block (ZrIr_10_) in the Cu_2_Mg-type phase, each Zr atom is surrounded by twelve Ir atoms in the *Cmmm* structure, resulting in a different polyhedron building block. By the full relaxations of both lattice constants and internal atomic coordinations, the equilibrium structural parameters of *Cmmm* structure are calculated to be *a* = 12.477 Å, *b* = 4.012 Å, and *c* = 3.946 Å, with four inequivalent atoms Zr, Ir1, Ir2, and Ir3 occupying 4*g* (0.653, 0, 0), 2*a* (0, 0, 0), 2*c* (0.5, 0, 0.5), and 4*g* (0.836, 0, 0.5) positions, respectively. Figure 1c presents the dependence of the total energy on the f.u. volume for *Cmmm* and Cu_2_Mg-type phases; it can be clearly seen that, for Ir_2_Zr, the predicted *Cmmm* structure is energetically far more stable than Cu_2_Mg-type structure, and this further confirms our structural prediction. Moreover, by fitting the third-order Birch-Murnaghan equation of state (EOS) [32] based on the calculated *E-V* data (Figure 1c), the zero-pressure bulk modulus (*B*_0_) and its pressure derivatives (*B*_0_′) of *Cmmm* phase is determined to be 235 GPa and 4.691, respectively. The dynamical stability of a crystalline structure requires the eigen frequencies of its lattice vibrations be real for all wave vectors in the whole Brillouin zone. As shown in Figure 1d, no imaginary phonon frequency was detected in the whole Brillouin zone for the predicted *Cmmm* phase, indicating its dynamical stability at ambient pressure.

**Figure 2:**
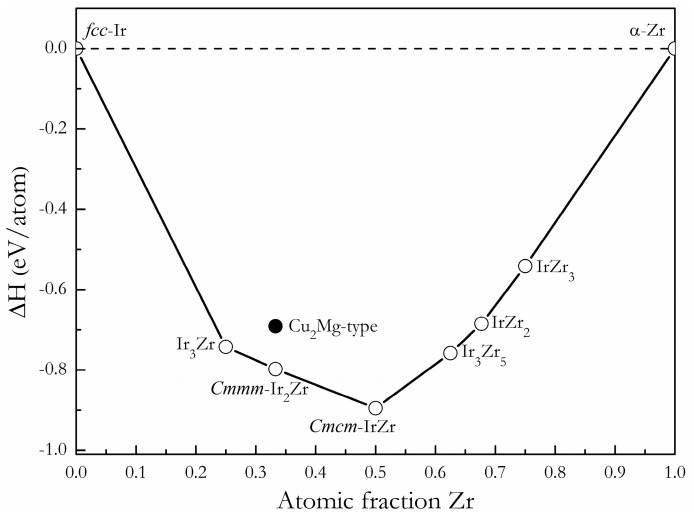
The formation enthalpy vs. composition curves for stoichiometric intermetallics in Ir-Zr system with *fcc*-Ir and *α*-Zr as reference states. The solid line denotes the ground state convex hull.

The formation enthalpy vs. composition plot, called a convex hull, is the set of lines connecting the lowest energy structures, and any structure whose formation enthalpy lies on the convex hull is deemed stable and synthesizable in principle [33]. In the Ir-Zr system, the formation enthalpy of each Ir*_x_*Zr*_y_* intermetallic with respect to the *fcc*-Ir and *α*-Zr separate phases is quantified by the formula. Based on the calculated formation enthalpy for each Ir*_x_*Zr*_y_* intermetallic compound, we reconstructed the convex hull line for the Ir-Zr system, as shown in Figure 2. One can see that the formation enthalpy of Ir-Zr intermetallic decreases when *x* < 0.5, while the formation enthalpy increases when *x* > 0.5. The *Cmcm*-IrZr is the most stable intermetallic compound among all the studied intermetallic compounds, which is in excellent agreement with the previous works [18]. The predicted *Cmmm* structure for Ir_2_Zr matches evidently with the convex hull curve between the experimental L1_2_-Ir_3_Zr and *Cmcm*-IrZr phases, indicating the possible synthesis of this composition in the real experiment. However, the previously proposed Cu_2_Mg-type phase for Ir_2_Zr is located above the convex hull line and possesses a higher formation enthalpy, at 107 meV/atom, than that of the *Cmmm* phase, suggesting that the Cu_2_Mg-type phase for Ir_2_Zr is indeed metastable. In addition, we suppose that the synthetic conditions of *Cmmm*-Ir_2_Zr may be similar to those observed for the formation of Ir_3_Zr_5_ for their similar values for formation enthalpies presented in the convex hull line.

As a new intermetallic phase in the Ir-Zr system, the mechanical properties of *Cmmm*-Ir_2_Zr are important for high-temperature applications. Table 1 and Table 2 present the calculated results on the mechanical parameters of *Cmmm*-Ir_2_Zr and Cu_2_Mg-Ir_2_Zr, including the single crystal elastic constants (*C_ij_*), polycrystalline elastic modulus, and hardness, which are determined from the calculated *C_ij_* by applying a set of given strains. Under the stress-strain approach [30], for a given set of strains ***ε*** = (*ε*_1_, *ε*_2_, *ε*_3_, *ε*_4_, *ε*_5_, *ε*_6_) (where *ε*_1_, *ε*_2_, and *ε*_3_ are the normal strains and others are the shear strains) imposed on a crystal, correspondingly, one set of stresses ***σ*** = (*σ*_1_, *σ*_2_, *σ*_3_, *σ*_4_, *σ*_5_, *σ*_6_) can be determined on the deformed lattice in terms of first-principles calculations; herein, the elastic stiffness constant matrix ***C*** links ***ε*** and ***σ*** by ***σ*** = ***ε C***. Based on the obtained elastic constants, bulk modulus *B* and shear modulus *G* are evaluated by using the Voigt-Reuss-Hill approximation [31], and the Young’s modulus *E* and Poisson’s ratio *v* are derived from the equations of *E* = 9*BG* = (3*B* + *G*) and *v* = (3*B* − 2*G*) = (6*B* + 2*G*), respectively. The empirical model of *H_v_* = 2(*k*^2^*G*)^0.585^ − 3 (*k* = *G*/*B*) proposed by Chen et al. [34] employed here to estimate the hardness of Ir_2_Zr. As listed in Table 1, the three independent *C_ij_* calculated for the cubic Cu_2_Mg-Ir_2_Zr are in excellent agreement with the previous theoretical results [18], confirming the reliability of the present results and the accuracy of the elastic constant calculations. The mechanical stability of the predicted *Cmmm* phase satisfies the Born-Huang criterion for an orthorhombic crystal [35]: (*C*_11_ > 0, *C*_44_ > 0, *C*_55_ > 0, *C*_66_ > 0, *C*_11_*C*_22_ > *C*_12_^2^, *C*_11_*C*_22_*C*_33_ + 2*C*_12_*C*_13_*C*_23_ − *C*_11_*C*_23_^2^ − *C*_22_*C*_13_^2^ − *C*_33_*C*_12_^2^ > 0), thus suggesting that the *Cmmm* phase is mechanically stable under ambient conditions. In addition, the calculated results for the *Cmmm* phase showed that the *C_ij_* possess the trend *C*_11_ ≈ *C*_22_ ≈ *C*_33_, suggesting that it is nearly the same uniaxial compression resistance along the three main crystal directions. Polycrystalline elastic modulus, another important parameter, also contains information regarding the hardness of a material with respect to various types of deformation. Bulk modulus *B* measures the resistance of a material to volume change and provides an estimate of its response to a hydrostatic pressure, shear modulus *G* describes the resistance of a material to shape change, and Young’s modulus *E* measures the resistance against uniaxial tension. From Table 2, firstly, it should be noted that the derived Hill bulk modulus *B* of *Cmmm* phase (239 GPa) agrees well with that obtained directly from the fitting of the Birch-Murnaghan EOS (*B*_0_ = 235 GPa), which further demonstrates the accuracy of our elastic constant calculations. Secondly, the calculated values of bulk modulus *B*, shear modulus *G*, Young’s modulus *E*, and hardness for *Cmmm* phase are close to those of Cu_2_Mg-type phase, indicating their similar polycrystalline mechanical behaviors. Thirdly, according to the Pugh criterion [36], the intrinsically brittle nature of both *Cmmm* and Cu_2_Mg-type structures for Ir_2_Zr can be revealed by its *B*/*G* ratio of 2.038 and 1.920, which is larger than the critical value of 1.75.

**Figure 3:**
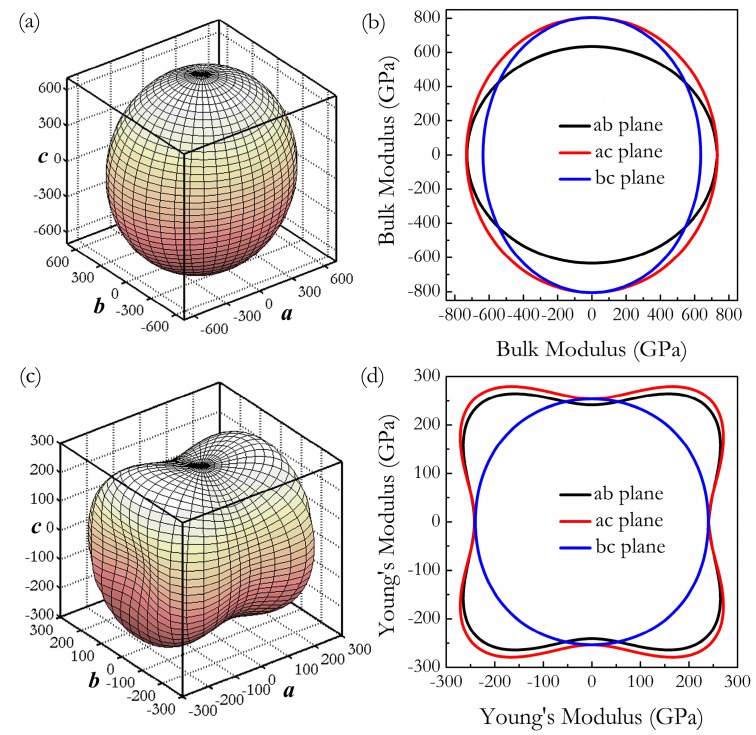
Three-dimensional surface representations of bulk modulus *B* (**a**) and the Young’s modulus *E* (**b**) at 0 GPa, and the projections of bulk modulus *B* (**c**) and the Young’s modulus *E* (**d**) within *ab*, *ac*, and *bc* planes.

**Figure 4:**
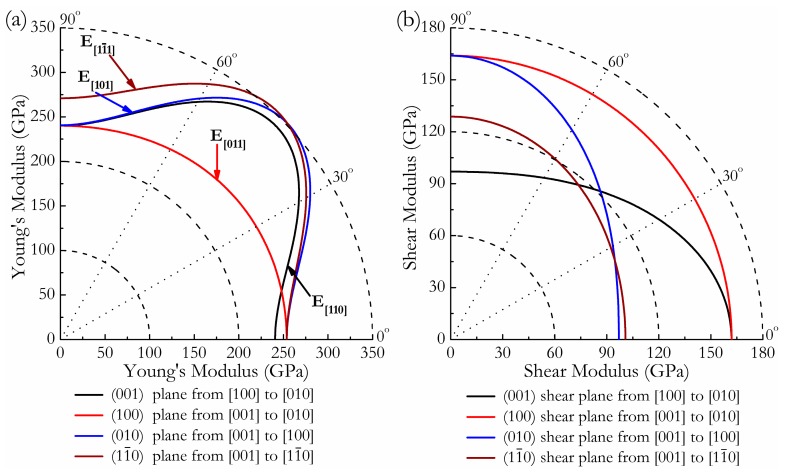
Orientation dependences of the Young’s modulus *E* (**a**) and shear modulus *G* (**b**) for *Cmmm*-Ir_2_Zr.

The elastic anisotropic property, which is strongly related to the mechanical strength of solid materials, has important implications in engineering applications, such as microcracks, anisotropic plastic deformation, elastic durability, etc. Compared to the reported results of other known intermetallic compounds in the Ir-Zr system, the studies on the elastic anisotropic behaviors of this new *Cmmm* phase are of great importance for its technical applications in high-temperature environments. As outlined by Panda et al. [37] and He et al. [38], executing the appropriate coordinate system transformations for the compliances allows the determination of the variation of bulk modulus *B*, Young’s moduli *E*, and shear modulus *G* with crystallographic direction, [*uvw*], for a given crystallographic plane, (*hkl*), containing these directions, (i.e., *B*_[*uvw*]_, *E*_[*uvw*]_, and *G*_(*hkl*)[*uvw*]_). For orthorhombic *Cmmm* phase, the bulk modulus *B* and Young’s modulus *E* can be expressed as:

(1) $$B^{-1}=(s_{11}+s_{12}+s_{13})\alpha^{2}+(s_{12}+s_{22}+s_{23})\beta^{2}+(s_{13}+s_{23}+s_{33})\gamma^{2}$$

(2) $$E^{-1}=s_{11}\alpha^{4}+s_{22}\beta^{4}+s_{33}\gamma^{4}+2s_{12}\alpha^{2}\beta^{2}+2s_{23}\beta^{2}\gamma^{2}+2s_{13}\alpha^{2}\gamma^{2}+s_{44}\beta^{2}\gamma^{2}+s_{55}\alpha^{2}\gamma^{2}+s_{66}\alpha^{2}\beta^{2}$$

where *α*, *β*, and *γ* are the direction cosines of [*uvw*] direction, and *s*_11_, *s*_22_, etc. are the elastic compliance constants given by Ney [39]. The shear modulus *G* on the (*hkl*) shear plane with shear stress applied along [*uvw*] direction is given by:

(3) $$G^{-1}=4s_{11}\alpha_{1}^{2}\alpha_{2}^{2}+4s_{22}\beta_{1}^{2}\beta_{2}^{2}+4s_{33}\gamma_{1}^{2}\gamma_{2}^{2}+8s_{12}\alpha_{1}\alpha_{2}\beta_{1}\beta_{2}+8s_{23}\beta_{1}\beta_{2}\gamma_{1}\gamma_{2}+8s_{13}\alpha_{1}\alpha_{2}\gamma_{1}\gamma_{2}\;+s_{44}{\left(\beta_{1}\gamma_{2}+\beta_{2}\gamma_{1}\right)}^{2}+s_{55}{\left(\alpha_{1}\gamma_{2}+\alpha_{2}\gamma_{1}\right)}^{2}+s_{66}{\left(\alpha_{1}\beta_{2}+\alpha_{2}\beta_{1}\right)}^{2}$$

where *α*_1_, *β*_1_, *γ*_1_, *α*_2_, *β*_2_, *γ*_2_ are the direction cosines of the [*uvw*] and [*HKL*] directions in the coordinate systems, where the [*HKL*] denotes the vector normal to the (*hkl*) shear plane. The three-dimensional (3D) surface representations showing the variation of the bulk modulus *B* and Young’s modulus *E* are plotted in Figure 3a,c, and the distance from the origin of the system of coordinates to this surface is equal to the *B* or *E* in a given direction. The plane projections (*ab*, *ac*, and *bc* planes) of the directional dependences of the bulk modulus *B* and Young’s modulus *E* are given in Figure 3b,d for comparison. It can be seen that *Cmmm*-Ir_2_Zr exhibits a highly pronounced elastic anisotropy, as its 3D picture shows a large deviation from the spherical shape, which qualifies an isotropic medium. From Figure 3b,d, the distributions of bulk modulus *B* and Young’s modulus *E* within the crystal plane *bc* display the largest and the smallest elastic anisotropy behaviors, respectively. In more detail, the changes of Young’s moduli along different crystal directions within four specific planes (001), (100), (010), and $\left(1\bar{1}0\right)$ are presented in Figure 4a. For example, the variation of Young’s modulus *E* in the (100) plane for the quadrant of directions [*uvw*] between [001] (*θ* = 0°) and [010] (*θ* = 90°), the *Cmmm* phase displays the smallest elastic anisotropy behavior (as shown in Figure 3d) with a maximum of 254 GPa and a minimum of 240 GPa. The selected directional Young’s moduli along the five principal crystallographic directions are denoted as in Figure 4a, and it can be seen that the calculated values for *E* decrease in the following order: *E*_[100]_ ≈ *E*_[010]_ < *E*_[011]_ < *E*_[001]_ < *E*_[110]_ < *E*_[110]_ < *E*_[101]_ < $E_{\left(1\bar{1}0\right)}$ < $E_{\left(1\bar{1}1\right)}$. Similarly, Figure 4b presents the orientation dependence of the shear modulus along arbitrary shear directions within these four shear planes. Compared to the other three shear planes, the calculations show that the shear modulus for a shear deformation within the (100) plane is not only nearly isotropic [*G_max_* = *G*_[010]_ = 164 GPa and *G_min_* = *G*_[001]_ = 162 GPa] but also has greater resistance to shear deformations.

**Figure 5:**
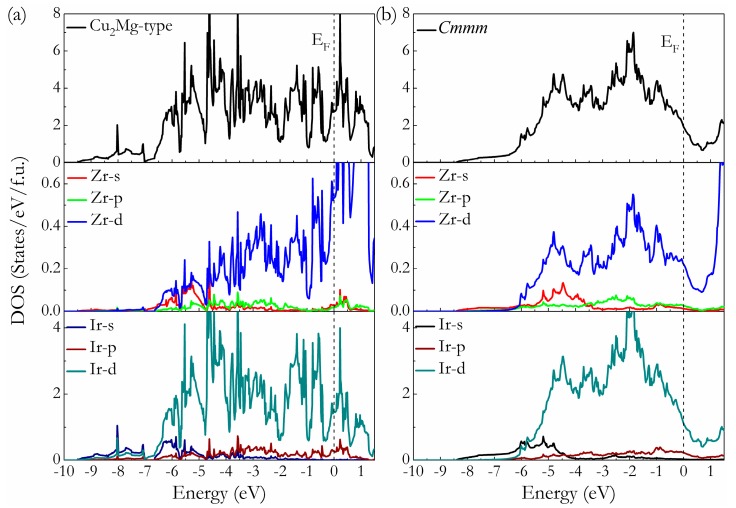
Calculated total and partial DOS of Cu_2_Mg-Ir_2_Zr (**a**) and *Cmmm*-Ir_2_Zr (**b**). The vertical dash line is the E_F_.

To understand the bonding mechanism of intermetallic Ir_2_Zr on a fundamental level, the total and partial density of state (*t*-DOS and *p*-DOS) of Cu_2_Mg-type and *Cmmm* phases at ambient pressure are calculated and presented in Figure 5, where the vertical dashed line is the Fermi level (E_F_). It is clear that both structures are metal for the finite values of *t*-DOS at the E_F_. The typical feature of the *t*-DOS for these two compounds is the presence of so-called “pseudogap” (a sharp valley around the E_F_), a borderline between the bonding and antibonding orbital [40]. It has been suggested that the location of E_F_ in the DOS profiles can reflect the structural stability of the compounds. For the Cu_2_Mg-type phase, the E_F_ lies to the right of the pseudogap (i.e., within the antibonding states), suggesting their metastable nature, while for *Cmmm* phase, the E_F_ locates at the left of the pseudogap (i.e., within the bonding states) with relative lower electronic density of state N(E_F_) values, revealing its structural stability. These findings support the previous results acquired from the formation enthalpy calculations. The feature of *p*-DOSs for both phases indicates that there is strong interaction between Ir and Zr atoms through *d*-*d* hybridization, which can be seen from the hybridization peaks of Ir-*d* and Zr-*d* orbitals plotted in Figure 5a,b, signifying the existence of directional covalent-like bonding in Ir_2_Zr intermetallic compound. A careful comparison further reveals that the *p*-DOS of the Ir and Zr atoms in *Cmmm*-Ir_2_Zr are more localized than those in Cu_2_Mg-Ir_2_Zr, resulting a small bandwidth of *p*-DOS and a lower N(E_F_). It is known that for the most stable structure, there is enough room to accommodate all its valence electrons into bonding states, so as to bring the E_F_ to a valley position separating bonding and antibonding states (pseudogap) favorable for structural stability. Therefore, the formation of *Cmmm* intermetallic phase is energetically more favorable than the previously proposed Cu_2_Mg-type for Ir_2_Zr.

## 4. Conclusions

In summary, we have extensively explored the ground-state structures of stoichiometric Ir_2_Zr intermetallic compound by using the recently developed particle swarm optimization algorithm in crystal structure prediction. A new orthorhombic *Cmmm* structure was proposed as the best candidate at ground state that is energetically more favorable than the previous experimental Cu_2_Mg-type structure, as demonstrated by the total energy and formation enthalpy calculations. Then, the mechanical and electronic properties of this new predicted *Cmmm* phase were fully investigated in comparisons with Cu_2_Mg-type phase. The predicted *Cmmm* phase exhibits nearly elastic isotropic behavior and great resistance to shear deformations within (100) crystal plane according to the directional elastic moduli calculations. Evidence of atomic bonding related to the structural stability for Ir_2_Zr was obtained by calculation of the electronic structures.

## Figures and Tables

**Table 1:** Calculated single elastic constants *C_ij_* (in GPa) of Cu_2_Mg-type and *Cmmm* phases for Ir_2_Zr intermetallic compound.

Ir_2_Zr	Source	*C*_11_	*C*_22_	*C*_33_	*C*_44_	*C*_55_	*C*_66_	*C*_12_	*C*_13_	*C*_23_
Cu_2_Mg-type	This work	379	-	-	152	-	-	181	-	-
-	Theory [18]	379	-	-	151	-	-	180	-	-
*Cmmm*	This work	362	348	378	97	162	164	173	188	174

**Table 2:** Calculated polycrystalline bulk modulus *B*, shear modulus *G*, Young’s modulus *E*, and hardness *H* (in units of GPa) for Ir_2_Zr. Also shown are Poisson’s ratio *v* and *B/G* ratio.

Ir_2_Zr	Source	*B*	*G*	*E*	*v*	*B/G*	*H*
Cu_2_Mg-type	This work	248	129	323	0.283	1.972	18.9
-	Theory [18]	246	128	327	0.278	1.920	20.3
*Cmmm*	This work	239	117	302	0.289	2.038	18.4
